# Symmetrical facial hyperpigmentation in a Hispanic woman

**DOI:** 10.1016/j.jdcr.2024.10.026

**Published:** 2024-11-17

**Authors:** Lucy Wang, Hasret Gunduz, Kenneth Shulman, Kenneth Helmandollar, Frederick Pereira, Mehmet Fatih Atak, Banu Farabi

**Affiliations:** a School of Medicine, New York Medical College, Valhalla, New York; b The Feinstein Institutes for Medical Research, Northwell Health, Manhasset, New York; c Dermatology Department, New York Medical College, Valhalla, New York; d Dermatology Department, NYC Health + Hospital/Metropolitan, New York, New York; e Dermatology Department, NYC Health + Hospital/Coney Island, Brooklyn, New York

**Keywords:** dermatoscopy, dermoscopy, Hori nevus, hyperpigmentation, melanocytic, nevus

## Case presentation

**Fig 1:**
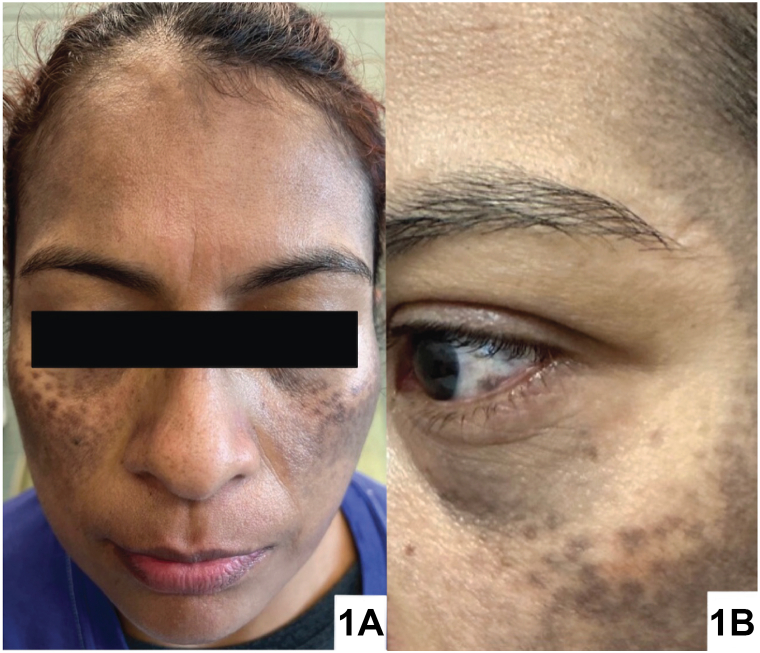

**Fig 2:**
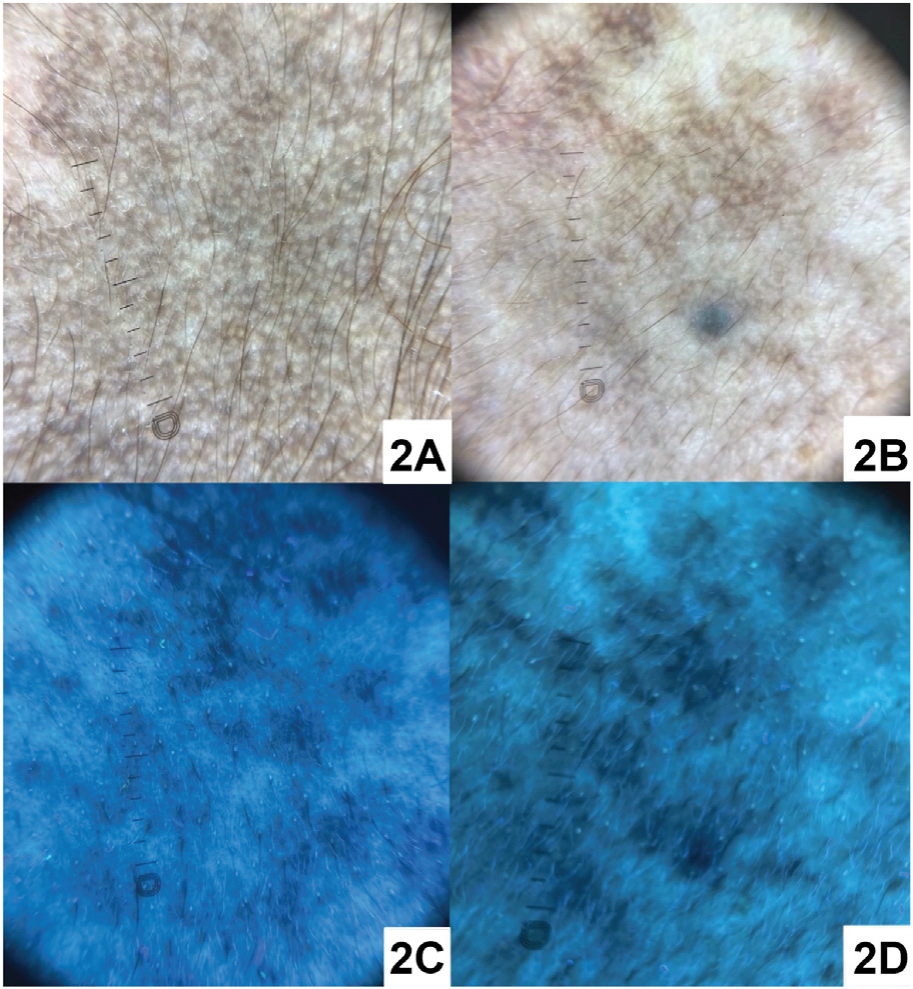

**Fig 3:**
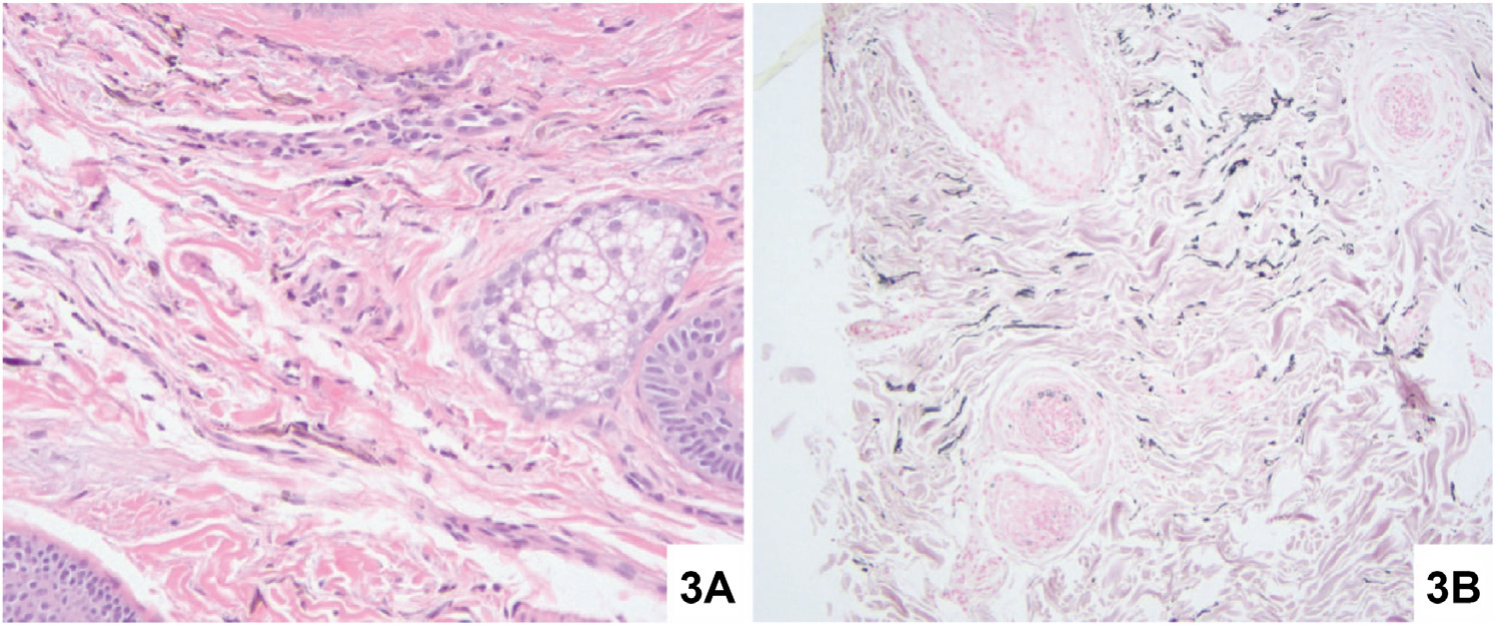

A 41-year-old Hispanic woman with Fitzpatrick skin type IV presented with a 10-year history of asymptomatic facial hyperpigmentation worsening over the past year without identifiable triggers. Topical hydroquinone and tretinoin treatment were unsuccessful. Physical examination revealed bilateral well-demarcated brown-gray hyperpigmented macules coalescing into patches on the temporal forehead, zygomatic cheeks, and nasolabial folds, and conjunctival pigmentation (Fig 1). Dermatoscopy and Wood’s lamp—showing few areas of enhancement, accentuation of blue-gray globules, and a reticulated appearance—were performed (Fig 2). A 3-mm punch biopsy was obtained from the left lateral cheek for histopathology and Fontana Masson staining (Fig 3).

**Question 1: What is the most likely diagnosis?**

A. Melasma
B. Exogenous ochronosis
C. Hori nevus (HN)
D. Riehl melanosis
E. Lichen planus pigmentosus (LPP)

**Answers:**

A. Melasma – Incorrect. Melasma predominantly affects Fitzpatrick skin types III to VI, presenting as symmetric brown facial hypermelanosis, but the lesions have irregular rather than well-defined borders.
B. Exogenous ochronosis – Incorrect. Exogenous ochronosis presents as brown-black macules over bony prominences of the face and neck, typically associated with long-term use of topical hydroquinone.^1^ While the patient had used topical hydroquinone, the exacerbated hyperpigmentation began before treatment.
C. Hori nevus (HN) – Correct. HN presents as discrete brown macules that gradually merge into confluent gray-brown patches. HN primarily affects patients with Fitzpatrick skin types III and IV, particularly Asian women.^2^ It commonly occurs on the bilateral malar regions but can also involve the forehead, temples, eyelids, and nasal ala and root. Scleral and conjunctival pigmentation have also been noted.^3^
D. Riehl melanosis – Incorrect. Riehl melanosis is characterized by diffuse brown-gray hyperpigmented macules and is associated with a type IV hypersensitivity reaction to contact allergens, especially cosmetics.^1^ This reaction is inconsistent with our patient’s history.
E. Lichen planus pigmentosus (LPP) – Incorrect. LPP is an uncommon variant of lichen planus that presents as brown, blue, or violaceous macules and patches symmetrically in sun-exposed or intertriginous areas and more rarely found in linear, follicular, or blaschkoid patterns.

**Question 2: Which dermatoscopic findings are most characteristic of this condition?**

A. Brown pseudonetwork pattern, telangiectasias, and sparing of follicular openings
B. Blue-brown or gray pigmentation and a speckled homogenous pattern
C. Pseudonetwork pattern, blue-gray to brown dots and globules, and telangiectasias
D. Starburst pattern and comet tails
E. Moth-eaten borders and fingerprinting

**Answers:**

A. Brown pseudonetwork pattern, telangiectasias, and sparing of follicular openings – Incorrect. These findings, along with arcuate and annular structures, are common in melasma but rarely seen in HN.^4^ Since melasma is the main differential diagnosis leading to misdiagnosis of HN, dermatoscopy can serve as a valuable noninvasive tool in diagnosis.
B. Blue-brown or gray pigmentation and a speckled homogenous pattern – Correct. Blue-brown or gray pigmentation and a speckled homogenous pattern are characteristic of HN.^4^ Dermatoscopic examination of our patient revealed a reticulated pattern of brown-gray pigmentation with involvement of follicular openings (Fig 2, *A*). In some areas, blue-gray globules were observed on a background of light brown-gray speckled pigmentation, resembling a Wagyu beef-like appearance (Fig 2, *B*).
C. Pseudonetwork pattern, blue-gray to brown dots and globules, and telangiectasias – Incorrect. Dots and globules with blue-gray to brown pigmentation in a pseudonetwork pattern are characteristic dermatoscopic features of LPP. Less commonly, telangiectasias may appear in LPP.^5^
D. Starburst pattern and comet tails – Incorrect. A starburst pattern and comet tails, also known as micro-Koebnerization, are findings observed specifically in progressive vitiligo lesions.^5^
E. Moth-eaten borders and fingerprinting – Incorrect. These features are unique to solar lentigines and are not present in HN.^5^

**Question 3: What histopathological findings are seen in this condition?**

A. Solar elastosis, increased vascularization, and an altered basement membrane
B. Irregularly shaped melanocytes in the upper and mid dermis
C. Inflammatory infiltrate at the dermal-epidermal junction
D. Banana-shaped yellow-brown pigmented lesions in the dermis
E. Elongated rete ridges and melanin deposition in lower epidermal levels

**Answers:**

A. Solar elastosis, increased vascularization, and an altered basement membrane – Incorrect. Solar elastosis and increased vascularization are not seen in HN. Moreover, the basement membrane is intact in HN. These findings are more indicative of melasma.^1^
B. Irregularly shaped melanocytes in the upper and mid dermis – Correct. Histopathology of HN would show irregularly shaped melanocytes in the upper and mid dermis with intact skin architecture.^2^ Histopathologic examination of our patient showed a normal epidermis and melanocytes with prominent pigmented dendritic processes in the superficial dermis (Fig 3, *A*). Immunohistochemistry of our patient’s sample included a Fontana Masson stain highlighting melanin in dendritic processes (Fig 3, *B*) and positive Melan-A and SOX10 immunostains (not pictured).
C. Inflammatory infiltrate at the dermal-epidermal junction – Incorrect. Inflammatory infiltrate is not seen in HN. When inflammatory infiltrate is present at the dermal-epidermal junction, lichenoid conditions like LPP should be suspected.
D. Banana-shaped yellow-brown pigmented lesions in the dermis – Incorrect. Banana-shaped yellow to brown ochronotic pigment in the dermis is characteristic of exogenous ochronosis.^1^
E. Elongated rete ridges and melanin deposition in lower epidermal levels – Incorrect. Thin, curved, and elongated rete ridges and hyperpigmentation in the lower epidermis is consistent with solar lentigines.^1^ HN is a dermal, not epidermal, melanocytic disorder.

## Conflicts of interest

None disclosed.
